# Preparation of Quercetin-Loaded Lipid Nanoparticle-Embedded Hydrogels and Stability Studies

**DOI:** 10.3390/molecules31142539

**Published:** 2026-07-22

**Authors:** Chatchapong Tangjidapichai, Sarin Tadtong, Chuda Chittasupho, Weerasak Samee

**Affiliations:** 1 Faculty of Pharmacy, Srinakharinwirot University, Nakhon Nayok 26120, Thailand; chatchapong@g.swu.ac.th (C.T.); sarin@g.swu.ac.th (S.T.); 2 Faculty of Pharmacy, Chiang Mai University, Chiang Mai 50200, Thailand; chuda.c@cmu.ac.th

**Keywords:** quercetin, nanoparticle, stability, anti-inflammatory, antioxidant, HPLC, docking

## Abstract

Quercetin, a plant-derived flavonoid with potent antioxidant and anti-inflammatory properties, is limited for topical use by its poor aqueous solubility, low bioavailability, and chemical instability. This research developed and validated a formulation-driven strategy to stabilize quercetin by encapsulating it in lipid nanoparticles (QLNs) and embedding these in a Carbopol hydrogel, providing comprehensive physicochemical and functional stability data. A fully validated HPLC-PDA assay was used to quantify quercetin in the nanoparticle–hydrogel matrix. In vitro bioactivity testing showed notable antioxidant activity (DPPH IC_50_ 6.84 ± 0.12 µg/mL; ABTS IC_50_ 4.04 ± 0.08 µg/mL; FRAP 301.46 ± 3.68 µg/mL) and an anti-inflammatory effect in LPS-stimulated RAW264.7 macrophages (1 µM quercetin reduced NO from 50.72 ± 2.00 µM to 41.57 ± 3.12 µM, *p* < 0.05). Forced degradation mapping across acidic, basic, oxidative, and photolytic conditions defined degradation pathways (complete loss in 1 N NaOH at 24 h; greater retention in 1 N HCl, 3% H_2_O_2_, and UV-254). QLN–hydrogel formulations remained physically and chemically stable through heating–cooling cycles and 180-day storage at multiple temperatures, retaining >90% quercetin and preserving antioxidant and anti-inflammatory activities (<10% reduction). These results establish a robust, application-ready approach for maintaining quercetin’s chemical integrity and bioactivity in topical formulations.

## 1. Introduction

Quercetin, a flavonoid abundantly present in fruits, vegetables, and medicinal plants, has garnered increasing attention for its potential benefits in topical applications, particularly owing to its antioxidant and anti-inflammatory properties. These characteristics render quercetin a promising candidate for formulations designed to enhance skin protection and facilitate healing. One of the primary mechanisms by which quercetin exerts its protective effects on the skin is through its antioxidant activity. Research has demonstrated that quercetin can significantly mitigate oxidative stress in keratinocyte cells exposed to UVB radiation. This protective effect is primarily attributed to quercetin’s ability to enhance the clearance of reactive oxygen species (ROS), thereby preventing cell death and promoting cell survival within cutaneous tissues (Zhu et al. [1]). The structural features of quercetin, particularly its hydroxyl groups, play an integral role in its capacity to neutralize free radicals, which is essential for combating oxidative damage induced by environmental stressors, including UV radiation [2]. In addition to its antioxidant properties, quercetin exhibits noteworthy anti-inflammatory effects that contribute positively to skin health. It has been shown to inhibit the expression of pro-inflammatory mediators and cytokines across various cell types, including macrophages. This action can help alleviate inflammatory responses associated with skin conditions, thus promoting both wound healing and tissue regeneration [3]. For instance, quercetin has been reported to impede the UV-induced degradation of collagen and other extracellular matrix components, which are critical for maintaining skin integrity and preventing photoaging [3]. Moreover, the topical application of quercetin has been investigated in various formulations to enhance its bioavailability and efficacy. Given its low solubility and permeability, innovative delivery systems such as ethosomes and transethosomes have been developed to facilitate the penetration of quercetin through the skin barrier [4]. These lipid-based carriers improve the stability and release profile of quercetin, thereby enhancing its effectiveness in topical applications. Additionally, quercetin has been integrated into nanofibrous wound dressings, which not only provide a physical barrier but also deliver quercetin’s antioxidant and anti-inflammatory benefits directly to the wound site [5,6]. In vivo evaluation of the topical anti-inflammatory effects of 1.3% pure quercetin in a murine model of ear edema induced by arachidonic acid (AA) and tetradecanoylphorbol-13-acetate (TPA) demonstrated no activity in the AA-induced model but significant inhibitory activity in the TPA-induced model [7]. Furthermore, studies have indicated that quercetin can enhance wound healing in models of diabetes when applied topically. In these investigations, quercetin ointment demonstrated a significant improvement in wound closure and a reduction in inflammation, highlighting its potential as a therapeutic agent for managing chronic wounds [8]. This beneficial effect is likely attributable to quercetin’s ability to modulate inflammatory responses and promote cellular proliferation and migration, both of which are vital processes in wound healing.

One of the primary advantages of utilizing lipid nanoparticles is their capacity to encapsulate hydrophobic compounds such as quercetin, thereby enhancing both its solubility and stability. Lipid nanoparticles, including solid lipid nanoparticles (SLNs) and nanostructured lipid carriers (NLCs), create a protective environment for quercetin, preventing degradation and enabling controlled release [9,10]. This encapsulation not only preserves the integrity of quercetin but also enhances its bioavailability, leading to more effective therapeutic outcomes. Quercetin exhibits poor aqueous solubility and low permeability, limiting its oral bioavailability [11]. Encapsulation within lipid-based nanocarriers improves their apparent solubility and facilitates enhanced absorption across biological membranes [12]. Studies have demonstrated that quercetin-loaded lipid nanoparticles can significantly improve its absorption and pharmacokinetics compared to free quercetin [13,14]. Lipid nanoparticle systems may enable dose reduction through enhanced delivery efficiency, thereby mitigating potential toxicity [15]. The controlled and sustained release from the nanoparticle matrix may prevent sharp plasma concentration peaks, thereby reducing the risk of adverse effects. Furthermore, the incorporation of quercetin into hydrogels significantly enhances its delivery system. Hydrogels are recognized for their biocompatibility and ability to retain moisture, making them ideal for topical applications. When quercetin is embedded in lipid nanoparticles within a hydrogel matrix, this combination facilitates sustained release and localized delivery of the flavonoid to target sites, such as in wound healing applications [16,17]. Sustained release is critical for maintaining therapeutic concentrations over extended periods, which is particularly advantageous in chronic wound management [18]. Additionally, the hydrogel matrix provides an optimal environment for cellular activities, promoting healing and tissue regeneration [19]. Moreover, the use of lipid nanoparticles in hydrogels can enhance the penetration of quercetin through biological barriers, including the skin and mucosal membranes. The lipid composition of the nanoparticles facilitates interactions with cell membranes, thereby enhancing the permeation of quercetin into the cells [20,21]. This characteristic is particularly relevant in dermatological applications, where effective skin penetration is essential for achieving desired therapeutic effects. Furthermore, the combination of quercetin with lipid nanoparticles and hydrogels can also modulate the release kinetics of the flavonoid. By varying the composition of the lipid nanoparticles and the hydrogel, researchers can tailor the release profile of quercetin to meet specific therapeutic needs [10,22]. This customization enables the optimization of treatment regimens, potentially resulting in improved patient outcomes.

An assay method for stability monitoring is essential for quantifying the active pharmaceutical ingredient (API) while concurrently assessing its stability under various conditions, including exposure to light, heat, and fluctuations in pH. Such methods are critical for ensuring compliance with regulatory standards and for maintaining the therapeutic efficacy of quercetin throughout its shelf life. For example, the method developed by Subramanian et al. successfully demonstrated the simultaneous estimation of quercetin and rutin under different stress conditions, including acidic, basic, and oxidative environments, thereby validating the robustness and reliability of the method for stability studies [23]. The application of High-Performance Liquid Chromatography with Photodiode Array (HPLC-PDA) detection allows for the precise quantification of quercetin by measuring its absorbance at specific wavelengths, which is advantageous due to the compound’s distinctive UV absorption spectrum. This method enables the quantitative monitoring of quercetin content under different stress and storage conditions. This capability is exemplified in the work of Kwak et al., who conducted HPLC analyses of quercetin glycosides, illustrating the method’s applicability in differentiating between various flavonoid derivatives [24]. Furthermore, the validation of the HPLC-PDA method entails the assessment of parameters such as specificity, linearity, accuracy, precision, and robustness, all of which are crucial for establishing the method’s reliability. For instance, the study conducted by Liu et al. highlighted the significance of method validation in guaranteeing that analytical results are reproducible and consistent, a fundamental requirement for quality control in pharmaceutical applications [25].

Quercetin, a flavonoid exhibiting antioxidant and anti-inflammatory properties, is susceptible to environmental degradation, necessitating rigorous stability assessment for therapeutic use. Here, a HPLC-PDA method was developed and validated for the quantification of quercetin in lipid nanoparticle-embedded hydrogels. Concurrently, quercetin-loaded lipid nanoparticles (QLNs) were formulated to enhance quercetin stability during manufacture and storage. The study aimed to preserve the quality, stability, and therapeutic efficacy of quercetin formulations. Additionally, the antioxidant and anti-inflammatory activities of quercetin were investigated using in vitro targeting TNF-α and iNOS to support its bioactivity.

## 2. Results

### 2.1. Antioxidant Activity of Quercetin

The evaluation of quercetin’s antioxidant activity revealed notable inhibitory effects, as presented in Table 1. Quercetin effectively inhibited DPPH, demonstrating an IC_50_ value of 6.84 µg/mL. Additionally, it showed the capacity to inhibit ABTS, yielding an IC_50_ value of 4.04 µg/mL. The Ferric Reducing Antioxidant Power (FRAP) was measured at 301.46 µg/mL, indicating a significant antioxidant capacity.

### 2.2. Anti-Inflammatory Activity of Quercetin

**Figure 1:**
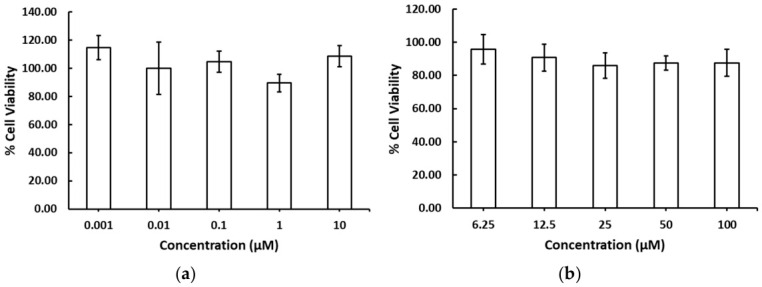
Percentage of cell viability of cultured RAW264.7 cells treated with (**a**) 0.001–10 µM quercetin (*n* = 3) and (**b**) 6.25–100 µM celecoxib (*n* = 3).

Quercetin showed no cytotoxicity on cultured RAW264.7 cells at all tested concentrations, varying from 0.001 to 10 µM. Quercetin at 0.001, 0.01, 0.1, 1 and 10 µM exhibited a % cell viability ± SD of 114.51 ± 8.54, 99.79 ± 18.61, 104.61 ± 7.52, 89.41 ± 6.36, and 108.53 ± 7.43, respectively (Figure 1a). Celecoxib was also tested at concentrations varying from 6.25 to 100 µM, and the results showed that no cytotoxicity of celecoxib was observed at any of the tested concentrations. Celecoxib at 6.25, 12.5, 25, 50 and 100 µM exhibited a % cell viability ± SD of 95.82 ± 8.86, 90.71 ± 8.07, 85.93 ± 7.65, 87.42 ± 4.24, and 87.58 ± 8.00, respectively (Figure 1b). Quercetin at 1 µM with celecoxib as a positive control at 50 µM was selected to be tested for its anti-inflammatory activity via observing its ability to reduce NO and TNF-α secretions against 1 µg/mL LPS-induced inflammation in RAW264.7 cells.

**Figure 2:**
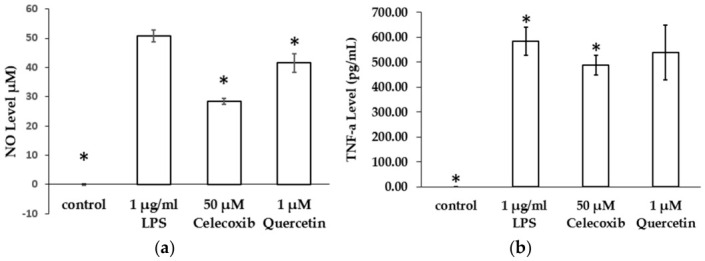
(**a**) NO level (µM) and (**b**) TNF-α level (pg/mL) of 1 µg/mL LPS-induced inflammation in RAW264.7 cells treated with 1 µM quercetin (*n* = 3) (* *p*-value < 0.05 compared with LPS-treated group).

Quercetin at 1 µM and celecoxib as positive control at 50 µM possessed interesting abilities to reduce NO secretion during 1 µg/mL LPS-induced inflammation in RAW264.7 cells. Quercetin at 1 µM significantly (*p* < 0.05) reduced NO secretion in the LPS-treated group from 50.72 ± 2.00 µM to 41.57 ± 3.12 µM, which was equal to an 18.04% reduction in the NO level, while the positive control, celecoxib, reduced NO secretion to 28.45 ± 1.04 µM, which was equal to a 43.97% reduction in the NO level (Figure 2a). Celecoxib at 50 µM significantly reduced the TNF-α secretions of the LPS-treated group from 595.46 ± 56.38 pg/mL to 488.83 ± 39.18 pg/mL, which was equal to a 16.51% reduction in the TNF-α level. Unfortunately, quercetin at 1 µM did not significantly reduce TNF-α secretions induced by 1 µg/mL LPS in RAW264.7 cells (538.48 ± 109.67 pg/mL, 8.03% reduction) (Figure 2b).

Quercetin at 1 µM exhibited anti-inflammatory activity via the reduction in NO secretions, which was related to its powerful antioxidation ability; however, this may not have been a proper concentration for reducing in TNF-α secretions.

### 2.3. Stability of Standard Quercetin in Force Degradation Conditions

The degradation study demonstrated that quercetin undergoes degradation, as evidenced by the decrease in peak areas compared to the corresponding peak areas of the initial quercetin solution at the same concentration, with no significant interfering peaks observed from degradation products, solvents, or stress reagents under the analytical conditions used. The percentage remaining was determined by comparing the areas of the quercetin peak under various degradation conditions with the area of the quercetin peak in non-degradation conditions. Forced degradation studies were conducted using 1 N HCl, 1 N NaOH, 3% *v*/*v* H_2_O_2_, and photodegradation at 2 h, 12 h, and 24 h.

**Figure 3:**
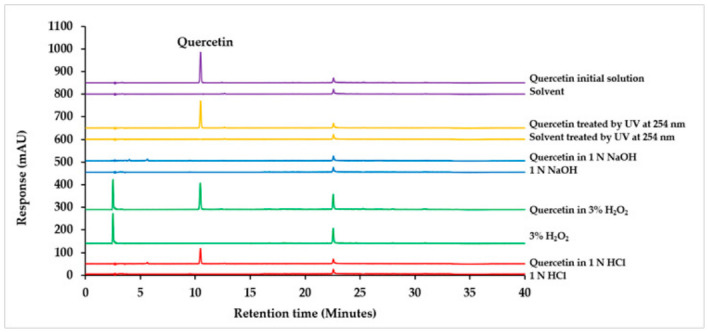
Chromatograms of quercetin from forced degradation studies conducted using 1 N HCl, 1 N NaOH, 3% *v*/*v* H_2_O_2_, and photodegradation for 2 h, with UV detection at 256 nm.

**Figure 4:**
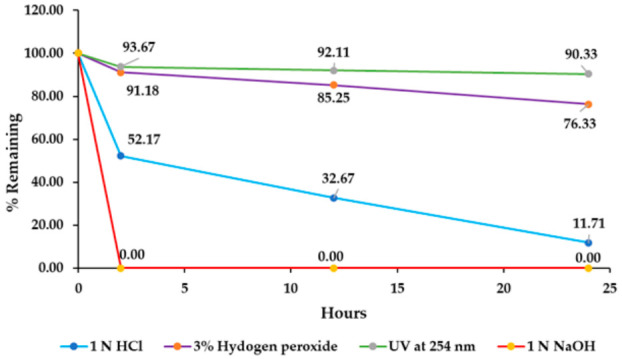
The percentage remaining profile of quercetin under forced degradation conditions, which include 1 N HCl, 1 N NaOH, 3% *v*/*v* H_2_O_2_, and UV light at 254 nm at intervals of 2 h, 12 h, and 24 h.

The quercetin peak was observed to disappear after 2 h in a 1 N NaOH solution, while a peak corresponding to approximately 50% of the original intensity remained in 1 N HCl. This observation indicates that the rapid degradation of quercetin occurs under alkaline conditions. In contrast, under neutral pH conditions, the quercetin peak remained detectable at approximately 90% intensity when exposed to UV light at 254 nm and 3% *v*/*v* H_2_O_2_ (Figure 3). The percentage remaining for the quercetin standard under these conditions ranged from 0% to 90%, as assessed using the developed HPLC method (Figure 4). A summary of the degradation studies for quercetin is presented in Table 2. Notably, quercetin demonstrated a higher rate of degradation in 1 N NaOH compared to 1 N HCl, 3% H_2_O_2_, and exposure to short-wavelength UV light (254 nm).

Based on the forced stability data for quercetin, it is recommended to formulate pharmaceutical preparations that maintain a pH within the range of 5.5 to 7.5. The gel formation process necessitates the use of a base, which could potentially compromise the stability of quercetin. Therefore, quercetin should be incorporated after the gel base has been prepared to prevent direct exposure to the base, thereby reducing the risk of degradation. Additionally, encapsulating quercetin in lipid nanoparticles may significantly improve its stability against environmental factors and the constituents of the gel formulation.

### 2.4. Physical Properties and Appearance of Quercetin-Loaded Lipid Nanoparticle and Hydrogels

**Figure 5:**
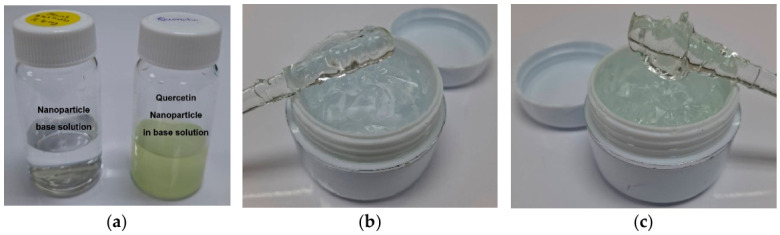

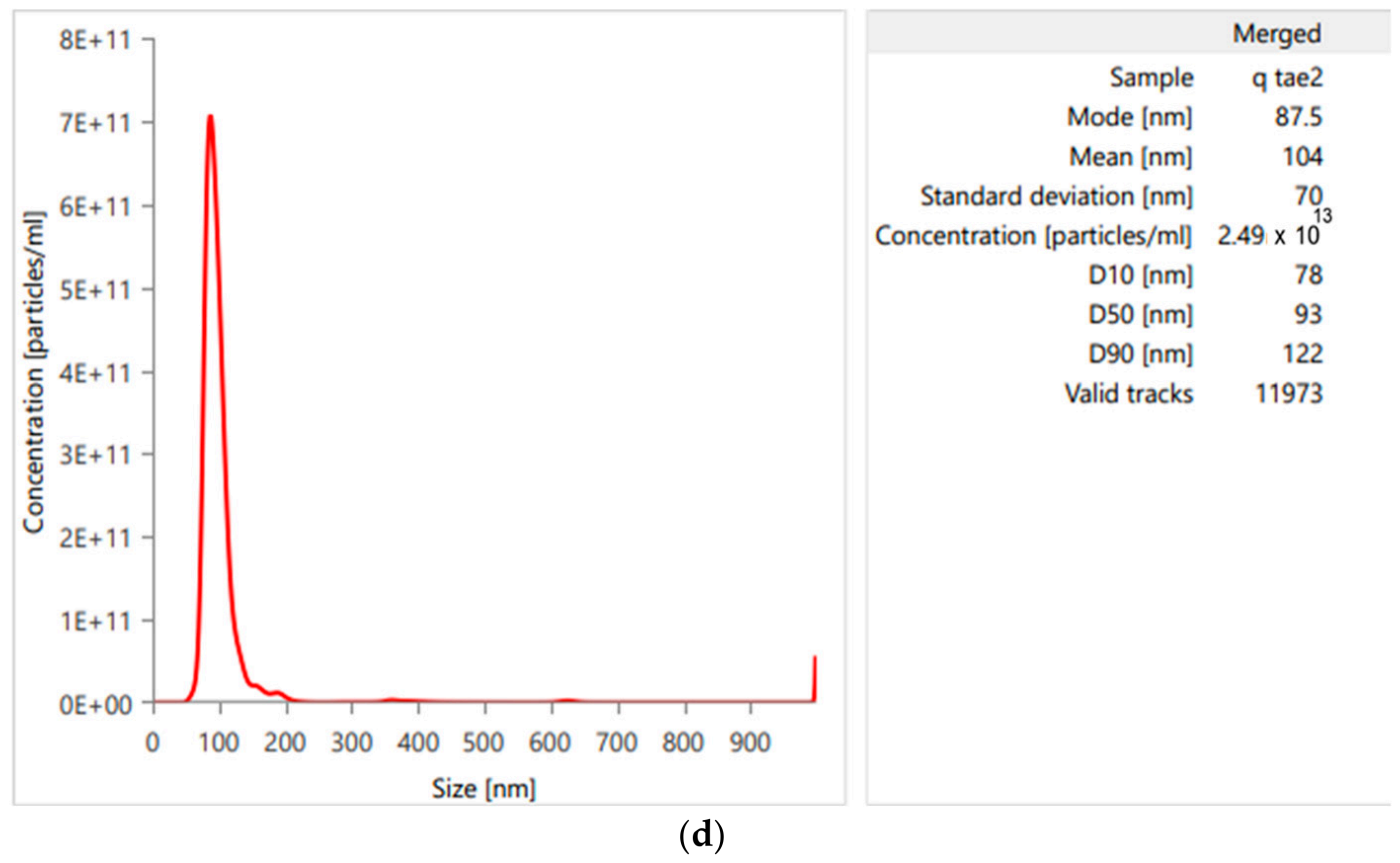
Images showing (**a**) quercetin nanoparticle solution, (**b**) gel base, (**c**) quercetin-loaded lipid nanoparticle-embedded hydrogels, and (**d**) particle size distribution of quercetin nanoparticles.

The data presented in Table 3 indicate that the quercetin-loaded lipid nanoparticles have an average particle diameter of 104.33 ± 2.85 nm (Figure 5d), with a particle size distribution (polydispersity index, PDI) of 0.115 ± 0.003 and a zeta potential of 0.0102 ± 0.0004 mV. The physical properties of the quercetin-loaded lipid nanoparticle-embedded hydrogels were characterized by a pH of 6.25 ± 0.11 and a viscosity of 117.42 ± 2.62 Pa·s. The appearance of the quercetin-loaded lipid nanoparticle-embedded hydrogels was observed to be a clear gel with a pale-yellow color, as illustrated in Figure 5.

### 2.5. Optimization of HPLC Method for Quantitative Analysis of Quercetin in Hydrogels and Stability Studies

**Figure 6:**
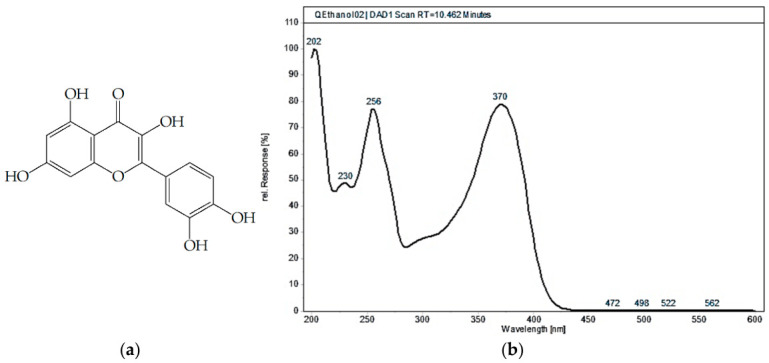
(**a**) Chemical structure and (**b**) UV spectrum of quercetin in mobile phase solution.

**Figure 7:**
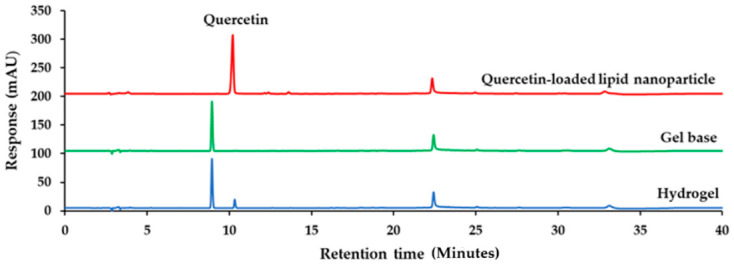
Chromatograms of quercetin nanoparticles, gel base, and quercetin-loaded lipid nanoparticle-embedded hydrogels, obtained with UV detection at 256 nm.

**Figure 8:**
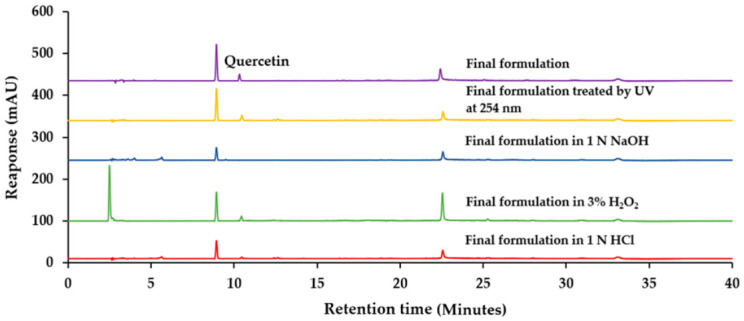
Chromatograms of quercetin-loaded lipid nanoparticle-embedded hydrogels from forced degradation studies conducted using 1 N HCl, 1 N NaOH, 3% *v*/*v* H_2_O_2_, and photodegradation for 2 h, with UV detection at 256 nm.

Based on the chemical structure of quercetin (Figure 6a), the UV–visible absorption spectrum within the range of 200–600 nm reveals two primary absorption bands: band A (240–280 nm) and band B (340–440 nm). In the mobile phase solution, quercetin exhibited maximum UV absorption at two wavelengths, i.e., 256 nm and 370 nm (Figure 6b). During the analysis of samples from forced degradation studies and quercetin-loaded lipid nanoparticle-embedded hydrogels utilizing the HPLC-PDA technique, the detector at 370 nm identified only the peak corresponding to quercetin at a retention time of 10.46 min, lacking information on degradation products or other components present in the hydrogel formulation. Therefore, 256 nm was selected for the quantitative monitoring of quercetin during stress and storage studies because it provided higher signal intensity and broader detection of UV-absorbing components in the formulation matrix (Figure 6 and Figure 7). Chromatograms from forced degradation studies showed progressive reduction in the quercetin peak intensity under stress conditions, while no distinct degradation product peaks were experimentally observed under the analytical conditions used (Figure 8). The chromatographic method and the choice of 256 nm are suitable for the stability study of quercetin, as they provide the specific detection of quercetin and adequate separation from degradation products and excipients.

### 2.6. Method Validation for Determination of Quercetin in Hydrogels and Degradation Samples

Quercetin, degradation products and other components present in the hydrogel’s formulation were successfully separated using a C18 HPLC column (250 × 4.6 mm, 5.0 µm). The mobile phase consisted of 0.1% formic acid in ultrapure water (phase A) and 0.1% formic acid in acetonitrile (phase B). The pretreatment sample was analyzed under the following gradient conditions. Initially, the gradient was adjusted from 20% to 80% of phase B over a 20 min period, maintained at 80% phase B for 10 min, returned to 20% phase B over the next 3 min, and held at 20% phase B for an additional 7 min. The analysis was conducted at a flow rate of 1 mL/min with a detection wavelength range of 200–600 nm, including specific wavelengths of 256 and 370 nm. The column temperature was maintained at 25 °C, and the injection volume was set at 20 µL. A quercetin peak appeared at retention times of 10.46 min, resulting in a resolution greater than 2.

The HPLC-PDA method was validated in accordance with AOAC guidelines with respect to linearity, limits of detection (LODs), limits of quantification (LOQs), precision, and accuracy.

Linearity was assessed using seven-point calibration curves over a concentration range of 0.5–50 µg/mL. The concentration levels were established based on the anticipated quantities of quercetin present in the degradation mixture and the hydrogel formulation, as informed by preliminary studies. Table 4 demonstrates that the coefficient of determination (R^2^), an indicator of linearity, exhibited exceptional linearity, yielding an R^2^ value of 0.9999. Additionally, the limits of detection (LOD) and limits of quantification (LOQ) were calculated from three calibration curves, resulting in values of 0.0099 µg/mL and 0.0450 µg/mL, respectively.

The recovery percentages for quercetin varied from 97.99% to 99.47%, as summarized in Table 5. Precision validation was assessed using the percentage relative standard deviation (%RSD). All %RSD values related to repeatability, as well as both intra-day and inter-day precision for the investigated markers, were found to be within 2.00%, indicating satisfactory results for precision validation (Table 5). This validated data confirms that the developed HPLC assay is an appropriate and accurate method for quantifying quercetin in both forced degradation samples and in the long-term stability studies of quercetin-loaded lipid nanoparticle-embedded hydrogel samples.

### 2.7. Stability Study of Quercetin-Loaded Lipid Nanoparticle-Embedded Hydrogels

**Figure 9:**
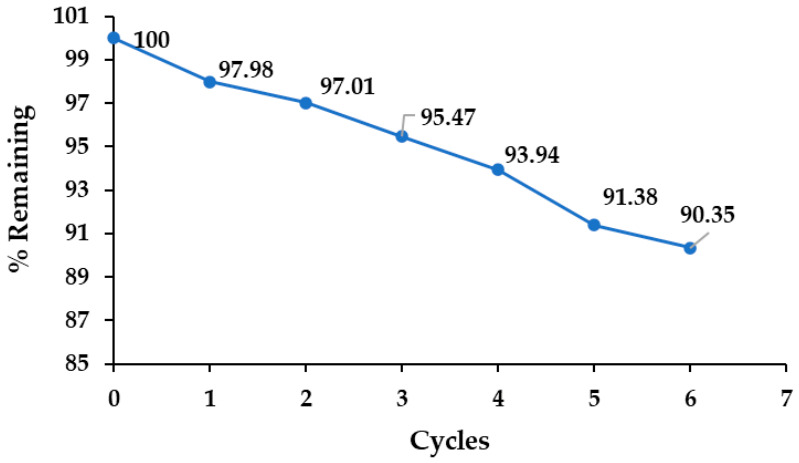
Heating–cooling stability study of quercetin-loaded lipid nanoparticle-embedded hydrogels.

**Figure 10:**
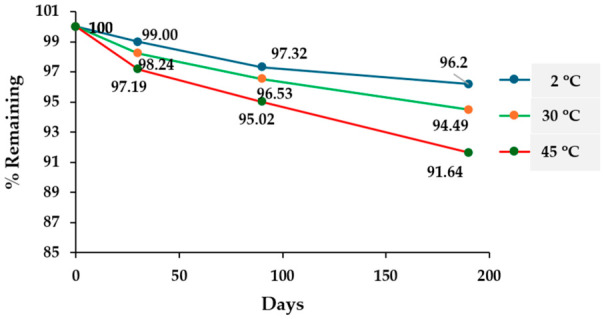
Storage stability study of quercetin-loaded lipid nanoparticle-embedded hydrogels in three storage conditions (2 °C, 30 °C, and 45 °C) at days 0, 30, 90, and 180.

Figure 9 and Table 6 illustrate the effects of temperature on the heating–cooling stability of quercetin-loaded lipid nanoparticle-embedded hydrogels, demonstrating a consistent decline in the remaining quercetin content with each cycle. After the sixth heating–cooling cycle, over 90% of the quercetin remained intact. As shown in Figure 10 and Table 7, under long-term storage conditions, increased temperatures adversely affected the remaining quercetin content. Notably, quercetin within the hydrogel exhibited considerable stability in both the heating–cooling stability study and during long-term storage across various temperatures, with percentage retention exceeding 90%. Maintaining appropriate storage conditions is essential for preserving quercetin content; therefore, this study recommends storing quercetin-loaded lipid nanoparticle-embedded hydrogels in a protective container at low temperatures.

### 2.8. Antioxidant and Anti-Inflammatory Activities of Quercetin-Loaded Lipid Nanoparticle-Embedded Hydrogels Before and After 180 Days of Storage at 45 °C

Antioxidant activity (Table 8): Quercetin incorporated into lipid nanoparticles (100 µg/mL) and quercetin-loaded hydrogels (200 µg/mL) exhibited pronounced radical-scavenging activity in both DPPH and ABTS assays, achieving approximately 97–99% inhibition. Following accelerated storage for 180 days at 45 °C, the hydrogels retained substantial antioxidant capacity, with a modest reduction to approximately 92.5% inhibition (DPPH: 92.55 ± 4.28; ABTS: 92.35 ± 4.01). The increased standard deviations after storage indicate greater variability, although overall antioxidant preservation remained high.

**Figure 11:**
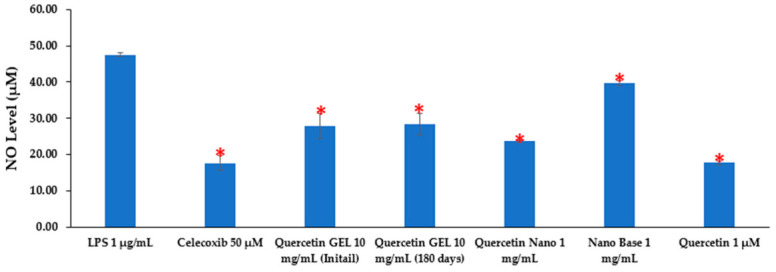
Nitric oxide (NO) levels (µM) in RAW 264.7 cells stimulated with 1 µg/mL LPS and treated with QNL hydrogels (10 mg/mL), before and after storage at 45 °C for 180 days (*n* = 3). * *p* < 0.05 vs. LPS-treated group.

Anti-inflammatory activity (Figure 11): In lipopolysaccharide (1 µg/mL)-stimulated RAW 264.7 macrophages, quercetin-loaded lipid nanoparticle (QNL) hydrogels (10 mg/mL) produced a significant decrease in nitric oxide production compared with the LPS-treated control (*p* < 0.05). Importantly, hydrogels subjected to 180 days of storage at 45 °C maintained a comparable and statistically significant suppression of NO, demonstrating preservation of anti-inflammatory efficacy under accelerated storage conditions.

Collectively, these data indicate that quercetin-loaded lipid nanoparticle hydrogels possess robust antioxidant and anti-inflammatory activities that are largely preserved after 180 days at 45 °C, with only a modest decline in antioxidant potency and slightly increased variability.

## 3. Discussion

This study demonstrates that quercetin possesses potent antioxidant activity and meaningful anti-inflammatory potential, and that encapsulation in lipid nanoparticles (QLN) within a hydrogel markedly enhances its chemical and functional stability for topical application. Quercetin exhibited a strong free-radical-scavenging and reducing capacity, with low IC_50_ values in DPPH (6.84 µg/mL) and ABTS (4.04 µg/mL) assays and a substantial FRAP value (301.46 µg/mL). These findings corroborate prior reports of quercetin’s robust antioxidant properties and support its suitability as an active ingredient in formulations designed to mitigate cutaneous oxidative stress [26].

In vitro anti-inflammatory activity was evidenced by a significant reduction in LPS-induced nitric oxide (NO) production in RAW264.7 macrophages at 1 µM, yielding a 18.04% decrease in NO without detectable cytotoxicity across the tested concentration range. The absence of a concomitant effect on TNF-α at this concentration suggests that, in this model, low-dose quercetin primarily modulates NO-related pathways (e.g., inhibition of iNOS or antioxidant-mediated suppression of reactive nitrogen species) rather than directly suppressing pro-inflammatory cytokine secretion. The greater inhibitory effect of the COX-2-selective inhibitor celecoxib on both NO and TNF-α highlights mechanistic and potency differences; dose–response experiments are therefore warranted to determine whether higher quercetin concentrations affect cytokine release [27,28,29].

**Figure 12:**
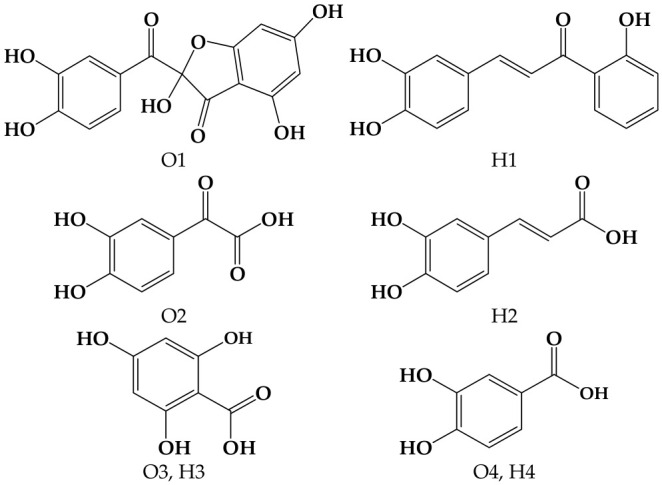
Proposed theoretical degradation pathways of quercetin (adapted from [30,31]).

Forced degradation studies revealed that quercetin is chemically labile under alkaline conditions, showing near-complete degradation in 1 N NaOH and partial degradation under acidic, oxidative, and photolytic stress. These results emphasize the importance of pH control and protection from oxidative and photolytic exposure during formulation and storage. Practically, the data support maintaining formulation pH in the mildly acidic to neutral range (~5.5–7.5) and avoiding strongly basic conditions during manufacture. Theoretically, and consistent with Chaaban et al. [30], oxidative degradation proceeds via the formation of O1 (2-(3′,4′-dihydroxybenzoyl)-2,4,6-trihydroxybenzofuran-3(2H)-one) and subsequent products O2–O4, while hydrolytic pathways yield H1–H4 (structures shown in Figure 12) [31,32]. However, these degradation products were not observed experimentally in the present study. Possible explanations for the absence of detectable degradant peaks include the formation of non-UV-absorbing species, conversion to highly polar products that coelute with the solvent front, complete mineralization, or the production of degradants at concentrations below the method’s LOD/LOQ.

The encapsulation of quercetin in lipid nanoparticles and incorporation into a hydrogel produced a colloidally stable, topically appropriate delivery system (mean particle size ≈ 104 nm, PDI 0.115, near-neutral zeta potential, gel pH 6.25) with rheological properties compatible with dermal application. The formulation’s physical characteristics and pale-yellow, clear appearance suggest likely acceptability for topical use and compatibility with skin physiology. The stabilization mechanism of the quercetin-loaded lipid nanoparticles is attributed to steric stabilization, primarily provided by the non-ionic surfactant (poloxamer 407), as well as the hydrogel matrix. Poloxamer 407 (PEO–PPO–PEO) forms a hydrophilic corona around nanoparticles, creating steric hindrance that prevents particle aggregation [33]. In addition, incorporation into the hydrogel network (Carbopol-based) further restricts nanoparticle mobility, effectively “trapping” the particles within a three-dimensional polymeric matrix, thereby enhancing physical stability despite the low surface charge.

Stability testing indicated that encapsulation substantially attenuates quercetin degradation: following heating–cooling cycles and 180 days of storage, >90% quercetin retention was observed alongside <10% loss of measured antioxidant and anti-inflammatory activities. QLN hydrogels retained a statistically significant suppression of LPS-induced NO production before and after storage (*p* < 0.05), indicating preserved pharmacological functionality. The concordance between retained antioxidant capacity and sustained NO inhibition supports a mechanistic link whereby maintained radical-scavenging and redox modulation contribute to preserved anti-inflammatory effects, consistent with encapsulation preserving bioavailable quercetin or active derivatives sufficient to elicit cellular responses [16,17,34,35].

Collectively, the results indicate that (1) quercetin exerts biologically relevant antioxidant and NO-modulating activity at low, non-cytotoxic concentrations; (2) quercetin is chemically unstable under alkaline, oxidative, and photolytic stresses; (3) QLN encapsulation within an appropriately formulated hydrogel substantially improves chemical and functional stability; and (4) the validated HPLC-PDA method reliably quantifies quercetin and its degradation products across diverse conditions.

This study has limitations, including the reliance on a single macrophage cell line and a limited cytokine panel for anti-inflammatory assessment, the absence of in vivo efficacy and skin-penetration data, and the lack of full structural elucidation of degradation products. Future work should include comprehensive dose–response analyses across a broader range of inflammatory mediators, evaluation of dermal absorption, bioavailability and in vivo efficacy, and detailed characterization of degradation pathways and products to inform long-term safety and regulatory assessment.

## 4. Materials and Methods

### 4.1. Chemicals and Reagents

The murine macrophage cell line RAW264.7 (ATCC TIB-71) was obtained from the American Type Culture Collection (Manassas, VA, USA). Dulbecco’s modified Eagle’s medium (DMEM, high glucose) and antibiotics–antimycotic solution were purchased from Gibco (Carlsbad, CA, USA). Fetal bovine serum (non-US origin), *N*-(1-naphthyl)ethylenediamine dihydrochloride, lipopolysaccharide (LPS), and celecoxib were supplied by Sigma-Aldrich (St. Louis, MO, USA). The CCK-8 assay kit was acquired from Dojindo (Nagasaki, Japan). Dimethyl sulfoxide (DMSO) and phosphoric acid were purchased from Merck (Darmstadt, Germany). A tumor necrosis factor-alpha (TNF-α) ELISA kit was obtained from AbClonal (Woburn, MA, USA). Sulfanilamide was purchased from Panreac (Barcelona, Spain), and sodium nitrite from Loba Chemie (Mumbai, India). Chromatography-grade acetonitrile and formic acid were obtained from Merck (Darmstadt, Germany), and quercetin was procured from Sigma-Aldrich (St. Louis, MO, USA).

### 4.2. In Vitro Antioxidant Activity Assay

#### 4.2.1. DPPH Radical-Scavenging Assay

The antioxidant capacity of quercetin was assessed using the DPPH (2,2-diphenyl-1-picrylhydrazyl) radical-scavenging assay, adapted from the protocol described in [26]. Briefly, 100 µL of the test solution was mixed with 100 µL of ethanolic DPPH solution (0.6 mM) and vortexed. The reaction was allowed to proceed for 30 min at room temperature in the dark, after which the absorbance was measured at 520 nm. A control containing DPPH solution without sample was included. Radical-scavenging activity (RSA) was expressed as percent DPPH discoloration and calculated as follows:

RSA (%) = [(Acontrol − Asample)/Acontrol] × 100%(1)

where Acontrol denotes the absorbance of the DPPH solution in methanol and Asample denotes the absorbance of the DPPH solution after addition of the quercetin sample. IC_50_ values, indicative of antioxidant potency, were determined through linear regression analysis. All measurements were performed in triplicate.

#### 4.2.2. ABTS Assay

The ABTS (2,2′-azino-bis(3-ethylbenzothiazoline-6-sulfonic acid)) radical cation scavenging activity of quercetin was assessed using a protocol adapted from [26]. The ABTS reagent was generated by mixing equal volumes of 7 mM ABTS and 2.45 mM potassium persulfate and incubating the mixture in the dark at room temperature for 24 h. The resulting ABTS stock was diluted with ethanol to an absorbance of 0.900 ± 0.020 at 735 nm prior to use. For each assay, 180 µL of the adjusted ABTS solution was combined with 20 µL of sample and incubated in the dark at room temperature for 30 min, after which absorbance was measured at 735 nm. The scavenging effect was expressed as percent inhibition:

ABTS scavenging effect (%) = [(Acontrol − Asample)/Acontrol] × 100%(2)

where Acontrol is the absorbance of the ABTS solution in methanol and Asample is the absorbance following addition of the sample. IC_50_ values, derived through linear regression, were used to quantify antioxidant capacity. All assays were performed in triplicate.

#### 4.2.3. Ferric Reducing Antioxidant Power (FRAP) Assay

The Ferric Reducing Antioxidant Power (FRAP) of quercetin was determined following the procedure of [26]. The FRAP reagent was prepared by mixing 25 mL acetate buffer (300 mM, pH 3.6), 2.5 mL TPTZ (10 mM in 40 mM HCl) and 2.5 mL FeCl_3_·6H_2_O (20 mM). In each assay, 20 µL of sample was combined with 180 µL of FRAP reagent and incubated in the dark for 30 min. The absorbance of the resultant blue Fe-TPTZ complex was measured at 595 nm. A calibration curve was generated using FeSO_4_ standards (170–2600 µM), and FRAP values were calculated through linear regression of the standards to express antioxidant capacity. All measurements were performed in triplicate.

### 4.3. In Vitro Anti-Inflammatory Activity Assay

#### 4.3.1. Cell Culture

RAW264.7 cells were cultured in high-glucose Dulbecco’s modified Eagle’s medium (DMEM) supplemented with 10% fetal bovine serum and 1% antibiotics–antimycotic, maintained at 37 °C in a humidified atmosphere containing 5% CO_2_. Monolayer cultures were subcultured every 3–4 days to preserve exponential growth.

#### 4.3.2. Cell Viability Assay

Cell viability was assessed using the CCK-8 assay. RAW264.7 cells were seeded in 96-well plates at 1 × 10^5^ cells/mL (100 µL/well) [36] and allowed to adhere for 24 h. The growth medium (high-glucose DMEM with 10% FBS and 1% antibiotics–antimycotic) was then replaced with a medium containing quercetin (dissolved in 0.5% DMSO) at final concentrations of 0.001, 0.01, 0.1, 1, and 10 µM. The medium containing 0.5% DMSO served as the vehicle control. Cells were incubated for 24 h at 37 °C, after which the treatment medium was removed and 100 µL of serum-free DMEM plus 10 µL CCK-8 reagent was added to each well. Following a 2 h incubation at 37 °C, absorbance was measured at 450 nm using a microplate reader. The results are reported as mean ± SD (*n* = 3, each in triplicate), with untreated cells cultured in complete medium defined as 100% viability. Percent cell viability was calculated accordingly.

% cell viability = (OD of treated cell/OD of untreated cell) × 100(3)

Quercetin concentrations that maintained cell viability >80% were selected for the subsequent evaluation of anti-inflammatory activity, assessed using measurements of nitric oxide (NO) and tumor necrosis factor-alpha (TNF-α) secretion.

#### 4.3.3. Anti-Inflammation Assay

RAW264.7 cells were seeded in 24-well plates at 5 × 10^5^ cells/mL (1000 µL/well). After 24 h, complete medium (high-glucose DMEM with 10% FBS and 1% antibiotics–antimycotic) was replaced with complete medium containing quercetin (dissolved in 0.5% DMSO) at concentrations that maintained >80% viability. Vehicle control consisted of 0.5% DMSO in complete medium; 50 µM celecoxib served as a positive control. Cells were pretreated for 1 h at 37 °C, after which lipopolysaccharide (LPS) was added to a final concentration of 1 µg/mL and incubation continued for 24 h at 37 °C (*n* = 3). Following incubation, culture supernatants were collected and stored at −80 °C until analysis of NO and TNF-α secretion.

##### Nitric Oxide Assay

Culture supernatants were assayed for nitrite using the Griess reaction, and absorbance was measured at 540 nm on a microplate reader. Nitrite concentrations were determined from a sodium nitrite standard curve. Briefly, 100 µL of each supernatant was dispensed into a 96-well plate and mixed with 100 µL Griess reagent (1% sulfanilamide and 0.1% N-(1-naphthyl)ethylenediamine dihydrochloride in 2.5% phosphoric acid), incubated for 10 min, and then read at 540 nm [36,37].

##### TNF-α Secretion Assay Using ELISA Kit

Tumor necrosis factor-alpha (TNF-α) levels in culture supernatants were quantified using a mouse TNF-α ELISA kit (AbClonal) following the manufacturer’s protocol. Briefly, 100 µL of appropriately diluted supernatant or TNF-α standards was added to a 96-well plate pre-coated with anti-mouse TNF-α and incubated at 37 °C for 2 h. Wells were then washed and incubated with 100 µL biotin-conjugated detection antibody at 37 °C for 1 h, followed by incubation with 100 µL streptavidin-HRP at 37 °C for 30 min. After a final wash, 100 µL 3,3′,5,5′-tetramethylbenzidine (TMB) substrate was added and incubated at 37 °C for 15 min, the reaction was terminated with 50 µL stop solution, and absorbance was read at 450 nm with reference at 570 nm.

### 4.4. Preparation of Quercetin-Loaded Lipid Nanoparticle

Quercetin-loaded nanoparticles were prepared through solvent displacement. An aqueous phase of 0.1% poloxamer 407 was prepared by dissolving 0.1 g poloxamer 407 in 100 mL deionized water. Quercetin (20 mg) was dissolved in 10 mL 95% ethanol and vortexed to ensure complete dissolution. Phosphatidylcholine was dissolved in 1 mL of the quercetin solution, and an additional 1 mL of 95% ethanol was added to yield a 2 mL organic phase. The organic phase was delivered from a syringe mounted on a syringe pump at 3 mL/h and injected dropwise into 15 mL of the aqueous phase under magnetic stirring (600 rpm) until complete addition, yielding quercetin-loaded lipid nanoparticles. Particle size, polydispersity index (PDI) and zeta potential were determined by dynamic light scattering (DLS) on a Zetasizer Nanoseries (Malvern Instruments, Worcestershire, UK) at 0.133 mg/mL, with a 173° scattering angle and at 25 °C. Nanoparticle tracking analysis (NTA) was performed on a NanoSight Pro (Malvern Panalytical, Worcestershire, UK) with a 488 nm laser and 20× objective. Samples were diluted 1:100 in particle-free water and introduced at 1.5 µL/min, and Brownian motion was recorded at ~30 fps; five recordings were acquired per sample. All measurements were carried out at room temperature, and data were processed using NS Explorer (NTA v1.2.0.3).

### 4.5. Preparation of Quercetin-Loaded Lipid Nanoparticle-Embedded Hydrogel

Distilled water (94.0 g) was placed in a beaker on a magnetic stirrer, and Carbopol 940 (1.0 g) was added gradually with continuous stirring until fully dispersed. Phenoxyethanol (1.0 g) was pre-dissolved in propylene glycol (5.0 g) and then incorporated into the Carbopol dispersion. Triethanolamine (0.1 mL) was added to neutralize and obtain a homogeneous hydrogel base. Finally, quercetin-loaded lipid nanoparticles (2.0 mL) were incorporated into the 100 g hydrogel and homogenized to ensure uniform distribution.

### 4.6. Force Degradation Study of Standard Quercetin

The forced degradation of quercetin was performed following ICH Q1A (R2) guidelines [38]. Quercetin (100 µg/mL) was subjected to acidic (1 N HCl), basic (1 N NaOH), neutral (water), oxidative (3% H_2_O_2_) and photolytic (UV, 254 nm) stress conditions. Aliquots were collected at 0, 2, 12 and 24 h; samples from acidic and basic treatments were neutralized prior to further handling. All samples were subsequently diluted with ethanol and analyzed using High-Performance Liquid Chromatography (HPLC).

### 4.7. Chemical Stability Study of Quercetin-Loaded Lipid Nanoparticle-Embedded Hydrogel

#### 4.7.1. Accelerated Stability Testing

Quercetin-loaded lipid nanoparticle-embedded hydrogels were equilibrated for 24 h and then subjected to an accelerated stability protocol comprising six thermal cycles, each consisting of 24 h at 45 °C followed by 24 h at 2 °C. After each cycle, hydrogel samples were collected and analyzed for quercetin content using HPLC.

#### 4.7.2. Storage Stability Study

Quercetin-loaded lipid nanoparticle-embedded hydrogels were stored protected from light at 2 °C, 30 °C, and 45 °C for up to six months. Samples were withdrawn at 0, 1, 3, and 6 months and analyzed for quercetin content using HPLC.

### 4.8. HPLC-UV Analysis

High-Performance Liquid Chromatography (HPLC) analyses were performed on an Agilent 1260 Infinity II system (Santa Clara, CA, USA) equipped with a quaternary pump, autosampler, column thermostat, and photodiode array detector. Separation was achieved on an ACE C18-AR column (4.6 × 250 mm, 5 µm; Avantor, Radnor, PA, USA) with a Phenomenex C18 guard column (4 × 3 mm, 5 µm; Torrance, CA, USA). The mobile phase consisted of 0.1% formic acid in ultrapure water (solvent A) and 0.1% formic acid in acetonitrile (solvent B). Samples were analyzed using a gradient program: 20 → 80% B over 20 min, hold at 80% B for 10 min, return to 20% B over 3 min, and re-equilibrate at 20% B for 7 min. The flow rate was 1.0 mL/min, column temperature 25 °C, and injection volume 20 µL. Detection was performed by PDA scanning from 200 to 600 nm, with monitoring at 256 and 370 nm.

### 4.9. Method Validation

The HPLC-PDA method was validated in accordance with ICH [38,39] and AOAC [40] guidelines, evaluating linearity, limit of detection (LOD), limit of quantification (LOQ), accuracy, and precision.

#### 4.9.1. Specificity

The specificity of the HPLC-PDA method was evaluated by comparing the chromatograms and UV spectra of mixed quercetin standards with those of the sample solutions at 370 and 256 nm. Peaks attributable to quercetin in the quercetin-loaded nanoparticle hydrogel and the hydrogel base were confirmed to be baseline-resolved from other chromatographic peaks based on retention time and spectral characteristics. Specificity was further corroborated by performing triplicate analyses of both standards and samples.

#### 4.9.2. Linearity

Seven-point calibration curves for quercetin were constructed over the concentration range 0.5–50 µg/mL, with each standard measured in triplicate.

#### 4.9.3. Limit of Detection (LOD) and Limit of Quantification (LOQ)

The limits of detection (LOD) and quantification (LOQ) were derived from the linear regression parameters using the following expressions:

LOD = 3.3 × (SD of the y-intercept)/(mean slope)

LOQ = 10 × (SD of the y-intercept)/(mean slope)

Thus, LOD and LOQ were calculated as 3.3- and 10-fold multiples, respectively, of the ratio between the standard deviation of the calibration curve intercept and the mean slope.

#### 4.9.4. Accuracy

Method recovery was assessed by spiking samples at three concentrations of quercetin (10, 20, and 30 µg/mL). Percent recovery was calculated by comparing measured concentrations to the nominal spiked amounts. Accuracy was expressed as the mean recovery (%) obtained from triplicate injections at the low, medium, and high levels.

#### 4.9.5. Precision

Method precision was evaluated through repeatability and intermediate precision. Repeatability was determined from three consecutive injections within a single day, while intermediate precision was assessed via triplicate injections on three separate days at low, medium, and high concentration levels. Precision was expressed as percentage relative standard deviation (%RSD).

### 4.10. Analysis of Quercetin-Loaded Lipid Nanoparticle-Embedded Hydrogels

One gram of quercetin-loaded lipid nanoparticle hydrogel was transferred to a centrifuge tube and extracted with 10 mL of 50% ethanol. The suspension was sonicated for 20 min and centrifuged at 8000 rpm and 5 °C for 10 min, and the supernatant was filtered through a 0.45 µm nylon membrane prior to HPLC injection.

### 4.11. Statistical Analysis

Data are presented as mean ± standard deviation (SD) from a minimum of three independent experiments. Statistical analyses were performed using GraphPad Prism version 8.0 (GraphPad Software, San Diego, CA, USA). Multiple group comparisons were evaluated using one-way ANOVA with Tukey’s post hoc test, whereas two-group comparisons employed unpaired *t*-tests. Differences were considered statistically significant at *p* < 0.05.

## 5. Conclusions

Quercetin’s potent antioxidant properties make it a promising ingredient for cosmetic and topical therapeutic formulations, but its chemical instability is a major formulation concern. The validated HPLC-PDA assay method enables the reliable quantification of quercetin and is suitable for stability monitoring within 40 min. Forced degradation studies showed rapid decomposition under alkaline conditions (1 N NaOH), partial degradation in acidic conditions (1 N HCl), and greater resistance to 3% H_2_O_2_ and short-wave UV exposure, although gradual loss occurred with prolonged stress. Encapsulation in lipid nanoparticles and incorporation into a hydrogel matrix, combined with maintaining formulation pH within a neutral range and protecting from oxidative and photolytic stress, markedly improve quercetin’s chemical stability and preserve biological activity. For optimal shelf life, storage at low temperatures is recommended.

## Figures and Tables

**Table 1:** Antioxidant activity of quercetin (IC_50_ ± SD, *n* = 3).

	DPPH (µg/mL)	ABTS (µg/mL)	FRAP Value FeSO_4_/mM (µg/mL)
Quercetin	6.84 ± 0.12	4.04 ± 0.08	301.46 ± 3.68

**Table 2:** Summary of degradation study of quercetin.

Force Degradation Conditions	% Remaining ± SD		
	2 h	12 h	24 h
Acid (1 N HCl)	52.17 ± 0.85	32.67 ± 1.91	11.71 ± 0.74
Alkali (1 N NaOH)	0.00 ± 0.00	0.00 ± 0.00	0.00 ± 0.00
Oxidation (3% *v*/*v* H_2_O_2_)	91.18 ± 1.04	85.25 ± 1.19	76.33 ± 2.24
Photolysis (UV at 254 nm)	93.67 ± 1.29	92.11 ± 1.94	90.33 ± 2.45

**Table 3:** Physical properties and appearance of quercetin-loaded lipid nanoparticles and hydrogels.

Quercetin-Loaded Lipid Nanoparticles				
Particle size diameter (nm)	PDI		Zeta potential (mV)	
104. 33 ± 2.85	0.115 ± 0.003		0.0102 ± 0.0004	
Quercetin-loaded lipid nanoparticle-embedded hydrogels				
Sample	pH	Viscosity (Pa·s)	Color	Clearness
Gel base	6.25 ± 0.11	117.42 ± 2.62	No	Clear
Gel base + nanoparticles	6.22 ± 0.14	116.35 ± 3.02	Pale yellow	Clear

**Table 4:** Linearity, LOD, and LOQ of quercetin at 256 nm UV.

Range (µg/mL)	Linear Equation	R^2^	LOD (µg/mL)	LOQ (µg/mL)
0.5–50	Y = 72.577X − 4.976	0.9999	0.0099	0.0450
	Y = 72.097X − 4.542	0.9999		
	Y = 72.720X − 4.769	0.9999		

**Table 5:** Accuracy and precision of quercetin (*n* = 3).

Day	Spiked (µg/mL)	Found (µg/mL)	% Recovery	SD	Intra-Day %RSD	Inter-Day %RSD
1	10.02	9.929	99.09	0.9745	0.98	1.01
2	10.05	9.961	99.11	1.0206	1.03	
3	10.04	9.909	98.17	1.0717	1.09	
1	20.04	19.725	98.43	1.2429	1.26	0.91
2	20.10	19.885	98.93	0.5866	0.59	
3	20.08	19.699	98.10	0.8551	0.87	
1	30.06	29.715	98.85	0.7016	0.71	0.95
2	30.15	29.990	99.47	0.7845	0.79	
3	30.12	29.515	97.99	0.8551	0.87	

**Table 6:** Percentage remaining of quercetin from heating–cooling stability studies of quercetin-loaded lipid nanoparticle-embedded hydrogels (Mean ± SD, *n* = 3).

	Cycle 0	Cycle 1	Cycle 2	Cycle 3	Cycle 4	Cycle 5	Cycle 6
% Remaining	100.00 ± 2.20	97.98 ± 3.73	97.01 ± 3.07	95.47 ± 2.99	93.94 ± 2.44	91.38 ± 0.97	90.35 ± 1.45

**Table 7:** Percentage remaining of quercetin from storage stability studies of quercetin-loaded lipid nanoparticle-embedded hydrogels in three storage conditions (2 °C, 30 °C, and 45 °C) at days 0, 30, 90, and 180 (Mean ± SD, *n* = 3).

Days	% Remaining		
	Storage at 2 °C	Storage at 30 °C	Storage at 45 °C
0	100.00 ± 2.20	100.00 ± 2.20	100.00 ± 2.20
30	99.00 ± 4.47	98.24 ± 2.27	97.19 ± 3.55
90	97.32 ± 3.86	96.53 ± 4.54	95.02 ± 2.86
180	96.20 ± 3.69	94.49 ± 3.18	91.64 ± 1.61

**Table 8:** Antioxidant activity of quercetin formulation before and after 180 days of storage (% inhibition ± SD, *n* = 3).

Sample	DPPH	ABTS
Quercetin nanoparticles (100 µg/mL)	98.56 ± 2.35	97.68 ± 2.76
Quercetin hydrogels (200 µg/mL)	97.75 ± 3.42	97.12 ± 3.37
Quercetin hydrogels after 180 days storage (200 µg/mL)	92.55 ± 4.28	92.35 ± 4.01

## Data Availability

The original contributions presented in this study are included in the article. Further inquiries can be directed to the corresponding author.

## Abbreviations

The following abbreviations are used in this manuscript:

ABTS	2,2′-Azino-bis(3-ethylbenzthiazoline-6-sulfonic acid)
AOAC	Association of Official Analytical Collaboration
C18	Octadecylsilane
°C	Celsius degree
cm	Centimeter
DAMPs	Damage-associated molecular patterns
DMSO	Dimethyl sulfoxide
DPPH	2,2-diphenyl-1-picrylhydrazyl
FRAP	Ferric Reducing Antioxidant Power
H_2_O_2_	Hydrogen peroxide
HCl	Hydrochloric acid
HPLC	High-Performance Liquid Chromatography
IC_50_	Half-maximal inhibitory concentration
ICH	The International Council for Harmonisation of Technical Requirements for Pharmaceuticals for Human Use
LPS	Lipopolysaccharide
mL	Milliliter
Mol	Mole
N	Normal
NaOH	Sodium hydroxide
NLCs	Nanostructured lipid carriers
Pa·s	Pascal second
pg	Picogram
QLNs	Quercetin-loaded lipid nanoparticles
RT	Retention time
SD	Standard deviation
ROS	Reactive oxygen species
SLNs	Solid lipid nanoparticles
TNF-α	Tumor necrosis factor-alpha
UV	Ultraviolet
VIS	Visible
µg	Micro gram
µM	Micro molar
%RSD	Percentage relative standard deviation
λmax	Wavelength of Maximum Absorbance
